# Autoimmune glial fibrillary acidic protein astrocytopathy misdiagnosed as intracranial infectious diseases: case reports and literature review

**DOI:** 10.3389/fimmu.2025.1519700

**Published:** 2025-01-22

**Authors:** Runhua Bai, Li An, Wei Du, Zhiwei Wang, Xiaokun Qi, Jianguo Liu, Ming Ren, Yingxin Yu

**Affiliations:** ^1^ Department of Neurology, The First Medical Center, Chinese PLA General Hospital, Beijing, China; ^2^ Graduate School of Inner Mongolia Medical University, Hohhot, China; ^3^ Department of Neurology, Beijing Jingxin Hospital, Beijing, China; ^4^ Department of Neurology, The Second affiliated Hospital, Anhui Medical University, Hefei, China; ^5^ Department of Neurology, Shanghai Blue Cross Brain Hospital, Shanghai, China

**Keywords:** Autoimmune glial fibrillary acidic protein astrocytopathy (A-GFAP-A), intracranial infection, meningitis, tuberculous meningitis(TBM), misdiagnose

## Abstract

**Background:**

Autoimmune glial fibrillary acidic protein astrocytopathy (A-GFAP-A) is an autoimmune central nervous system(CNS) disease characterized by GFAP IgG as a biomarker. Several cases of individuals with A-GFAP-A initially misdiagnosed as infectious diseases of the central nervous system have been reported in research. We report three cases of A-GFAP-A misdiagnosed as viral meningitis or tuberculous meningitis (TBM). We summarize recent cases of A-GFAP-A misdiagnosed as central nervous system infections through a literature review.

**Materials and methods:**

Three cases of A-GFAP-A were initially misdiagnosed as tuberculous or viral meningitis. Their diagnoses of A-GFAP-A were confirmed with positive GFAP-IgG in cerebrospinal fluid (CSF). We searched the PubMed database with the key words of “GFAP astrocytopathy”, “GFAP autoimmunity”, “GFAP autoantibody”, “intracranial infection”, “meningitis”, “misdiagnose”, and within the literature from Jan 1, 2015 to Mar 15, 2024, 40 cases with A-GFAP-A with positive GFAP-IgG in CSF who were previously misdiagnosed with intracranial infection were reported. The causes of misdiagnoses were summarized and analyzed.

**Results:**

Case 1 was a 41-year-old female, presenting with headache, fever, diplopia, and altered consciousness level. Anti-tuberculosis treatment was ineffective. Finally, with positive GFAP IgG in CSF, she was diagnosed with A-GFAP-A. Case 2 was a 74-year-old male, presenting with fever, excessive sweating, fatigue, and memory loss. Anti-tuberculosis treatment was ineffective. With positive GFAP IgG in CSF, he was diagnosed with A-GFAP-A. Case 3 was a 54-year-old male, presenting with fever, personality changes, and memory decline. Antiviral treatment was ineffective. His diagnosis was revised to A-GFAP-A after testing positive for GFAP IgG in CSF. Our study summarized a total of 40 patients with A-GFAP-A who were initially misdiagnosed as intracranial infections. The most common clinical phenotypes among 40 patients were mimicked meningitis, meningoencephalitis, meningoencephalomyelitis, encephalitis and encephalomyelitis.

**Conclusion:**

A-GFAP-A is a specific autoimmune meningoencephalomyelitis associated with GFAP-IgG, with lesions involved the brain, meninges, and spinal cord. It commonly presents with symptoms such as fever, headache, altered consciousness, tremor, seizures, and autonomic dysfunction. Brain MRI often shows characteristic linear perivascular radial enhancement perpendicular to the ventricles or nonspecific leptomeningeal enhancement. Early detection of GFAP-IgG in serum and CSF is essential for differential diagnosis.

## 1 Introduction

Autoimmune glial fibrillary acidic protein astrocytopathy (A-GFAP-A) is an immune-mediated inflammatory disease of the central nervous system associated with GFAP IgG antibodies. The brain, meninges, and spinal cord can be involved, with clinical manifestations such as fever, headache, changes in level of consciousness, tremors, seizures, and autonomic dysfunction. Patients may show characteristic linear radial perivascular enhancement perpendicular to the ventricles on brain MRI (1–4). This disease is a highly heterogeneous central nervous system syndrome with diverse clinical manifestations that mimic various clinical diseases. It is often misdiagnosed as other diseases such as CNS infectious diseases, demyelinating diseases, or tumors (5–7). Previous studies indicate that the misdiagnosis rate of autoimmune GFAP astrocytosis ranges from 4.5% to 35.7% (2, 8). This disease generally responds to immunotherapy, including corticosteroids, intravenous immunoglobulin, and immunosuppressants (9). Most patients have a good prognosis, some patients are prone to recurrence, and a few patients may have residual functional impairment or even death (10).

GFAP, an intermediate filament protein predominantly expressed in astrocytes, plays a key role in the pathogenesis of A-GFAP-A, although the exact mechanism remains unclear. GFAP plays a crucial role in regulating the morphology and movement of astrocytes, synaptic function, maintaining blood-brain barrier integrity, and normal CNS myelin formation (11). The anti-GFAP antibody targeting astrocytes is GFAP IgG, which is currently recognized as a specific biomarker in the CSF of patients with A-GFAP-A. It is usually detected through cell-based testing (CBA) or tissue-based testing (TBA) (1). Although anti-GFAP antibodies are present in the CSF of most patients, the non-pathogenicity of these antibodies remains controversial. It is hypothesized that the disease is autoimmune in nature, involving CD8+ cytotoxic T cells. The GFAP antibody serves solely as a biomarker of inflammatory changes and does not induce pathological changes itself (2, 12).

In recent years, there have been increasing reports on this disease. The non-specific symptoms of A-GFAP-A frequently lead to misdiagnosis and inappropriate treatment, especially during the early stages of the disease. In this paper, we reviewed the clinical data of three cases of A-GFAP-A diagnosed at the Sixth Medical Center of the General Hospital of the People’s Liberation Army, and conducted a literature review. We conducted a preliminary investigation into the similarities and differences in clinical manifestations, laboratory tests, and brain imaging manifestations in A-GFAP-A, as well as the reasons for misdiagnosis caused by A-GFAP-A, in order to improve clinicians’ understanding of this disease.

This study was approved by the Ethics Committee of the Sixth Medical Center of the General Hospital of the People’s Liberation Army (NO. HZKY2024-45).

## 2 Case presentation

### 2.1 Case 1

The patient was a 41-year-old female, who was admitted due to “19 days of headache with fever and 7 days of compromised consciousness level”. On the morning of June 26, 2020, the patient experienced severe bilateral temporal pain, accompanied by high fever. After 7 days of antibiotic treatment, she did not improve. On July 8th, compromised consciousness level and cognition were noticed, and the patient could not recognize family members. She also had nausea, vomiting, urinary retention, and constipation. On July 10th, a lumbar puncture was performed, showing a pressure of 320 mmH_2_O, white blood cells of 100 × 10^6^/L (92% lymphocytes, 12% eosinophils), protein level of 1.39 g/L, and glucose level of 1.5 mmol/L (blood glucose of 5.3 mmol/L), chloride of 107 mmol/L (blood chloride 94 mmol/L). Despite treatment for viral meningitis, her symptoms continued to worsen and she developed diplopia. Combining the patient’s clinical symptoms, imaging, and laboratory tests led the local hospital to consider TBM as a possibility, and gave anti-tuberculosis treatment with the addition of 10 mg/day of dexamethasone. On July 15th, the patient’s symptoms gradually improved, although intermittent low-grade fever persisted. She was transferred to our hospital on the same day for further treatment. The patient was previously healthy with no history of drug allergies or family history of genetic disorders. Neurological examination upon admission: drowsiness, cognition and thought within normal limit, and fine horizontal nystagmus. Limb muscle strength grade IV in four limbs, involuntary shaking of limbs, weakened bilateral tendon reflexes, right Babinski sign (+), neck stiffness with resistance, a 3-finger gap between the chin and chest, and an mRS score of 3.

**Figure 1:**
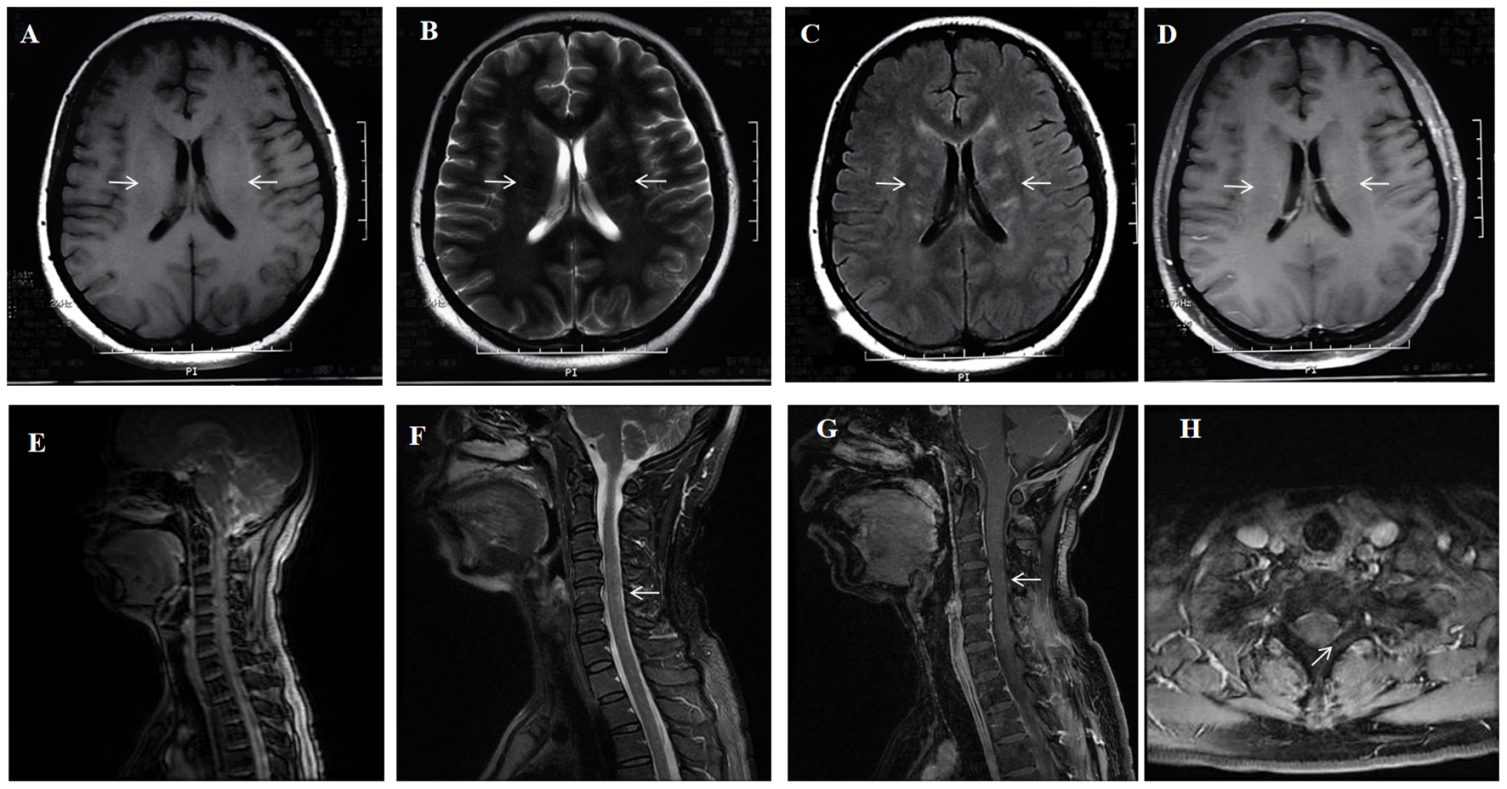
A 41-year-old woman who had no previous illness was admitted to our hospital complaining of fever and headache for 19 days and consciousness disturbance for 7 days. Brain MRI showed bilateral periventricular T1 hyposignaling **(A)** Brain MRI revealed T2 hyperintense lesions in the periventricular white matter **(B)** A corresponding FLAIR sequence showed hyperintense signals (arrows) in the periventricular white matter **(C)** Brain Contrast-enhanced T1-weighted (arrows) showed linear, radial perivascular pattern of enhancement **(D)**. Spinal MRI sagittal T2 showed diffuse multiple patchy and punctate hyperintensities in the cervical spinal cord and thoracic cord **(E, F)** Enhanced MRI of the cervical spinal cord showing point-flake diffuse enhancement in the cervical spinal cord **(G, H)**.

On July 19th, as the patient’s condition slowly worsened again, a repeat lumbar puncture revealed a pressure of 165 mmH_2_O, blood cells of 40 × 10^6^/L, white blood cells of 76 × 10^6^/L (99% lymphocytes), protein level of 0.513 g/L, and glucose level of 2.1 mmol/L (blood glucose 6.11 mmol/L), chloride of 115 mmol/L (blood chloride 95.2 mmol/L).The patient was evaluated for possible TBM, infectious diseases, autoimmune encephalitis, and paraneoplastic tumors, but all tests were negative. Aquaporin 4 (AQP4), myelin oligodendrocyte glycoprotein (MOG), myelin basic protein (MBP IgG), and autoimmune encephalitis-related antibodies were negative. Brain MRI showed abnormal high signals in the bilateral thalami, basal ganglia, cerebral peduncles, lateral ventricles, centrum semiovale, and subcortical areas, with perivascular radial enhancement around lateral ventricles (**Figures 1A–D**); Spinal MRI showed multiple abnormal high signals in the cervical and thoracic spinal cords (**Figures 1E–H**). On August 6th, a cell-based assay (CBA) for anti-GFAP IgG showed CSF GFAP IgG of 1:32 (+) and serum GFAP IgG of 1:32 (+). The diagnosis of A-GFAP-A was established. Daily intravenous immunoglobulin (0.4g/kg) and methylprednisolone 240 mg for one week resulted in significant improvement in the patient. Two weeks later, a repeat brain and spinal cord MRI with contrast enhancement showed a decrease in abnormal signals in the brain, cervical cord, and upper thoracic cord. After discharge, the patient was put on a taper of oral methylprednisolone. When methylprednisolone was reduced to 16mg, numbness in the four limbs occurred, and the numbness in both lower limbs worsened after long-distance walk. The symptoms improved with oral treatment of methylcobalamin and vitamin B1 tablets. The patient’s steroids were gradually tapered and discontinued, and treatment continued for about 3 months. Repeat brain and spinal cord MRI with enhancement after 3 months showed: scattered abnormal signals in the brain, with reduced range of lesions; The scattered abnormal signals in the cervical spinal cord were similar as before. No abnormal signals were found in the thoracic spinal cord. Five months after treatment, the CSF GFAP IgG remained 1:32 on repeat examination. The patient was followed up until March 2024, during which her condition remained stable with no recurrence, and her mRS score was 0. Due to the patient’s good recovery, the GFAP antibody titer and spinal cord MRI were not rechecked thereafter.

### 2.2 Case 2

The patient was a 74-year-old male, who was admitted due to “poor appetite, fatigue, low-grade fever for more than 3 months, upper limb shaking for 1 month, and lower limb tremors for 1 week”. The patient started to experience decreased appetite and fatigue in mid-March, 2021, then low-grade fever, excessive sweating and sleep in early May. On May 30th, he noticed involuntary tremors in both upper limbs, which gradually progressed. On June 15th, the patient began to experience memory difficulties. A lumbar puncture showed that a pressure of 110 mmH_2_O, CSF was pale yellow in color, with a white blood cell count of 120 × 10^6^/L (89.9% for mononuclear cells and 10.1% for multinucleated cells) and a protein level of 0.96 g/L. Combining the patient’s clinical symptoms, imaging manifestations and CSF characteristics, the local hospital considered TBM as a possibility. Administration of anti-tuberculosis therapy and low-dose dexamethasone treatment, but the symptoms did not improve. On June 24th, he developed involuntary tremors in both lower limbs. He had a history of chronic atrophic gastritis, with no history of drug allergy or familial genetic predisposition. Neurological examination upon admission: Hypofunction of higher cortex. Fine horizontal nystagmus, involuntary twitching of both orbicularis muscle, and right-sided neurogenic hearing loss. Increased distal muscle tone in both upper limbs, involuntary limb tremors, ataxia, Romberg sign (+), strong grasping and sucking reflex (+), hyperactive bilateral tendon reflex hyperactivity, and right Chaddock sign (+).

**Figure 2:**
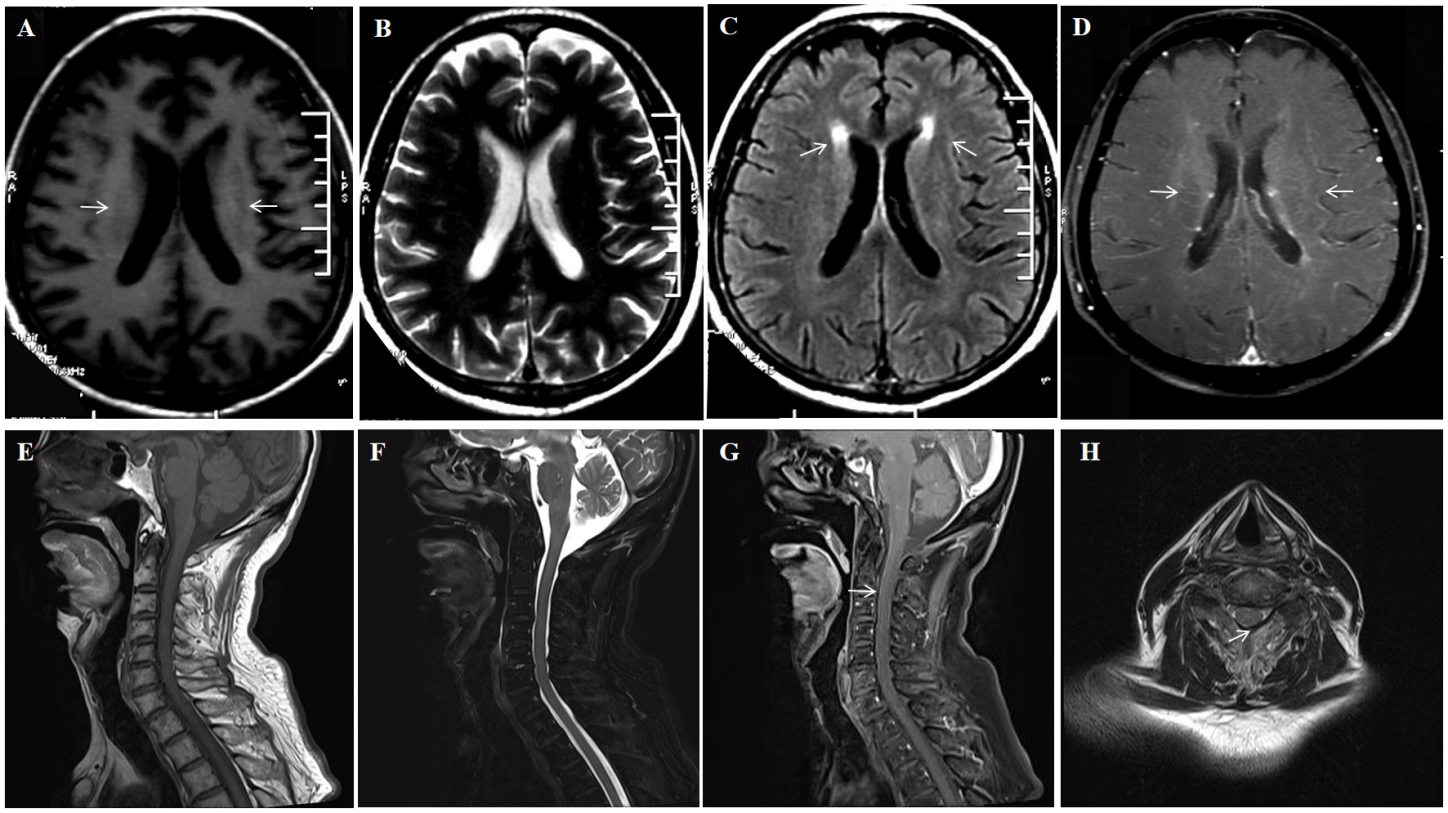
A 74-year-old man who was admitted to our hospital complaining of malaise and fatigue for more than 3 months, shaking of both upper limbs for 1 month and shaking of both lower limbs for 1 week. Brain MRI showed bilateral periventricular T1 hyposignaling **(A)** No abnormal signal is found on the T2 sequence **(B)** Brain MRI showed bilateral axial FLAIR hyperintensities (arrows) in the periventricular white matter **(C)** Brain Contrast-enhanced T1-weighted (arrows) showed multiple enhancements in the periventricular **(D)**. Spinal cord MRI T1 and T2 sequences did not show significant abnormal signals **(E, F)** Enhanced MRI of the cervical spinal cord (arrows) showed cervical medullary surface linear enhancement **(G, H)**.

After admission, contrast-enhanced brain MRI showed multiple abnormal enhanced lesions in the brainstem, bilateral temporal lobes, occipital lobes, local pia mater of the cerebellar hemisphere, right frontal lobe, left hippocampal sulcus and gyrus, and bilateral basal ganglia (**Figures 2A–D**). Contrast-enhanced MRI of the entire spinal cord showed silk-like enhancement in the cervical, thoracic, and lumbar spinal cords, as well as the filum terminale (**Figures 2E–H**). Electroencephalogram: diffused slow waves, more pronounced in the right hemisphere. PET/CT: Abnormal elevated glucose metabolism in bilateral basal ganglia, bilateral temporal lobes (with left hippocampus as the focus), partial temporal sulci, and lateral cerebral fissure meninges; uneven elevated glucose metabolism in the spinal cord. The bone marrow puncture results showed no abnormalities. A repeat of lumbar puncture revealed a pressure of 80 mmH_2_O, white blood cells of 70 × 10^6^/L (95% lymphocytes), and protein at 1.256 g/L. Consider the possibility that this patient may have TBM, infectious disease, autoimmune encephalitis, demyelinating disease, and paraneoplastic tumors. However, pathogen studies including tuberculosis, bacteria, and viral infections, were negative in CSF tests. Aquaporin-4 (AQP 4), myelin oligodendrocyte glycoprotein (MOG), myelin basic protein (MBP IgG), and autoimmune encephalitis-related antibodies were negative. Cell-based assay (CBA) showed CSF GFAP IgG of 1:100 (+), and A-GFAP-A was diagnosed. The patient was treated with 500mg methylprednisolone sodium succinate and 250mg mycophenolate mofetil, which resulted in significant relief of involuntary limb tremors and improved appetite, with an mRS score of level 2 two weeks later. Three months later, repeat brain and spinal cord MRI with enhancement showed scattered abnormal signals in the brain, with reduced range of lesions; The scattered abnormal signals in the cervical spinal cord were similar as before; No abnormal signals were found in the thoracic cord. Due to the fluctuating nature of the patient’s symptoms with each reduction of methylprednisolone tablets to 8 mg/day, which manifested as recurrent cognitive decline, the patient was maintained on long-term oral methylprednisolone tablets at 12 mg/day and morphine teicoplanin capsules at 250 mg twice daily. In August 2024, the patient’s symptoms worsened, with an mRS score of 3 and a CSF GFAP IgG level of 1:100. Brain MRI showed multiple white matter high signals in the more cerebral areas, significantly more than before.

### 2.3 Case 3

The patient, a 54-year-old male, was admitted to the hospital due to “2 months of fever, and one months of limb shaking, personality changes, and memory loss “. The patient developed low-grade fever after a cold in May 2021. On June 4th, he started to experience involuntarily shaking in both hands, changes in personality and preferences, incorrect answers to questions, memory decline, and emotional lability (crying easily). EB virus in serum was positive, and brain MRI showed a small ischemic lesion in the left parietal lobe. After antiviral treatment for viral meningitis, the patient’s symptoms did not improve. On June 26th, the patient’s body temperature returned to normal, but he started to experience balance problem, and feel stepping on cotton, and involuntary twitching of both lower limbs. The symptoms gradually worsened. On July 2nd, the patient developed excessive sleep, and constipation. The patient was previously healthy with no history of drug allergies family history of genetic disorders. Neurological examination upon admission: Hypofunction of higher cortex, uncontrolled limb tremors, ataxia, Romberg sign (+), bilateral hyperreflexia of tendons, bilateral palmar jaw reflex (+), bilateral Hoffman, Rossolimo, Babinski signs (+).

**Figure 3:**
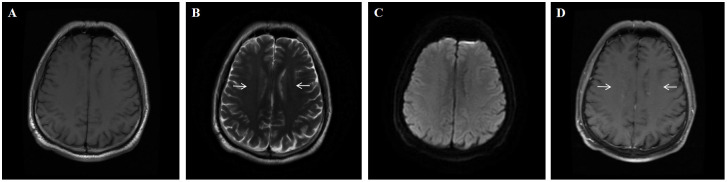
A 54-year-old man who had no previous illness was admitted to our hospital complaining of fever for 2 months, headache for 19 days and limb shaking, personality change and memory loss for more than 1 month. No abnormal signal is found on the T1 **(A)** and DWI **(C)** sequence; Brain MRI showed T2-weighted (arrows) hyperintensities in the periventricular white matter **(B)** Brain Contrast-enhanced T1-weighted (arrows) showed linear, radial perivascular pattern of enhancement **(D)**.

Upon admission, brain MRI showed scattered small patchy T_2_/FLAIR hyperintensities near bilateral cerebral ventricles, with abnormal vascular-like enhancement in bilateral periventricular and 4^th^ ventricular areas (**Figures 3A–D**). Contrast-enhanced spinal MRI showed no significant enhancement. Electroencephalogram showed diffuse slow waves on all leads. PET-CT showed diffuse decreases in glucose metabolism in the brain. Lumbar puncture showed a pressure of 110 mmH_2_O, white blood cells of 33 × 10^6^/L (100% lymphocytes) and protein at 0.807 g/L, glucose level of 3 mmol/L (blood glucose 7.5 mmol/L), chloride of 118mmol/L (blood chloride 98.7 mmol/L), and positive oligoclonal bands. Consider the possibility that this patient may have TBM, infectious disease, autoimmune encephalitis, demyelinating disease, and paraneoplastic tumors. However, pathogen studies, including tuberculosis, bacterial, and viral infections were negative in the CSF tests. Autoimmune encephalitis and paraneoplastic syndromes related antibodies were negative. Cell-based assay (CBA) revealed CSF GFAP IgG of 1:100 (+) and serum GFAP IgG of 1:100 (+), confirming the diagnosis of A-GFAP-A. 1000mg methylprednisolone and 250mg mycophenolate mofetil capsules were administered for the patient. After 2 weeks, the patient’s symptoms such as memory, unstable walking, and limb tremors improved significantly, but the improvement in mood and cognition were not significant. The patient was discharged and continued taking oral methylprednisolone 40 mg/day and morphine teicoplanin capsules 250 mg twice daily, with the methylprednisolone dose reduced by 4 mg every 10 days until discontinued. At his last follow-up (34 months after discharge), he was doing well without recurrence, but mild limb tremors remained, with an mRS score of 1. Unfortunately, the patient did not have his GFAP antibody titer and spinal cord MRI reviewed thereafter.

## 3 Literature review

We searched all English references published in the PubMed database between January 1, 2015 and March 15, 2024, using search terms (or combinations of these search terms) such as “autoimmune glial fibrous acidic protein astrocytopathy”, “GFAP astrocytopathy”, “GFAP autoimmunity”, “GFAP autoantibody”, “intracranial infection”, “meningitis” and “misdiagnosis”. Excluding articles without original data, 23 case reports and 5 case series were selected, including the 3 patients we report here. Finally, the demographic data, clinical features, CSF findings, brain imaging, treatment outcomes, and prognosis of 40 patients with A-GFAP-A initially misdiagnosed as intracranial infections were summarized and analyzed (**Table 1**).

**Table 1:** Summary of clinical features of A-GFAP-A patients initially diagnosed with CNS infection.

Case	Author	Age/sex	Initial symptoms	Main clinical symptoms	T2-FLAIR hyperintensities	CE-T1WI	CSF WBC/μL (lym%) protein, mg/L	Pri	Tre	Pro
1	Quek et al. (13)	56/M	fever, headache	confusion, hiccups, tremor	DWM/P, BS, cerebellum	LME	W:151 (92%) P: 1500	TBM	Steroids MMF	+
2	Quek et al. (13)	43/M	fever, neck stiffness, confusion	myelitis, seizure, hiccups	Us	LME	W: 287 (89%) P: 2370	TBM	Steroids	+
3	Quek et al. (13)	59/F	fever, headache	confusion, vomit	Us	LME	W:505 (91%) P: 1190	TBM	Steroids	+
4	Tajiri et al. (14)	23/M	fever, fatigue, headache	neck stiffness, dysuria, cognitive decline	bilateral parietal and temporal lobes	PLE	W:75(100%) P: 2390	CNSIs	Steroids	+
5	Ip et al. (15)	48/F	fever, neck stiffness, confusion	quadriparesis	left posterior temporo-occipital, BS, LETM	DSE	W:185(70%) P: Us	CNSIs	Steroids IVIG, LPEX rituximab	+
6	Du et al. (16)	19/M	fever, confusion, neck stiffness	dysurian, hiccups, fatigue	bilateral thalamus, DWM/P, BS	Us	W:108(90.7%) P: 1100	ME	Steroids, IVIG, LPEX	+
7	Duet et al. (16)	50/M	fever, headache	paraparesis, dysuria	cervical cord	Us	W:62(87.1%) P: 1500	ADEM	Steroids LPEX	+
8	Natori et al. (17)	47/M	fever, headache	confusion, fatigue	DWM/P	PLE LME	W: Ns P: 1400	HSVE	Steroids	+
9	Lin et al. (18)	58/M	fever, headache, confusion	neck stiffness, Paraparesis, dysuria, seizure	corpus callosum	LME	W: Us P: 158.71	TBM	Steroids MMF	+
10	Equiza et al. (19)	20/M	fever, headache, fatigue	neck stiffness, hyperekplexia, confusion	right thalamus, BS, BG	Us	W:56(85%) P: 1520	TBM	Steroids IVIG, LPEX rituximab	+
11	Bartels et al. (20)	33/M	fever, headache, quadriparesis	blurred vision, dysuria, cognitive decline	LESCLs	Us	W:154(Us) P: Us	MEM	Steroids MTX	+
12	Greco et al. (21)	33/F	fever, cough	headache, blurred vision	N	N	W:96(Us) P: Us	CNSIs	Steroids LPEX	+
13	Greco et al. (21)	19/F	fever, headache	blurred vision	DWM/P	PLE	W:45(Us) P: 640	CNSIs	Steroids	+
14	Canissario et al. (22)	31/M	headache, blurred vision	gait unsteadiness, symptoms of psychosis	bilateral optic nerves	Us	W:142(90%) P:1280	meningitis	Steroids LPEX, CTX	+
15	Lowe et al. (23)	44/M	fever, headache, vomit	fatigue, dysuria, confusion, tremor hallucinations	Leptomeningeal, BG, LESCLs	LME	W:400(94%) P:1960	meningitis	Steroids rituximab	+
16	Yang et al. (24)	40/M	fever, headache, vomit	neck stiffness, dysuria, agitate	BS	PLE	W:225(94%) P:2050	meningitis	Steroids	+
17	Yang et al. (24)	23/F	fever, headache	neck stiffness	DWM/P	PLE	W:300(97%) P:1550	meningitis	Steroids	+
18	Héraud et al. (25)	26/F	fever, headaches, diplopia, fatigue	walking difficulties, dysuria	callosum, cervical cord	LME	W:121(85%) P:1510	MEM	Steroids	+
19	Toledano et al. (26)	54/M	cognitive decline	headache, hallucinations, confusion	DWM/P, central cord	PLE CCE	W: (Us) (LRCHL) P:↑	prion diseases	Steroids CTX	+
20	Heide et al. (27)	45/F	back pain, fever, tremor	headache dysuria, cognitive decline, fatigue	Us	PLE	W:104(97%) P:1040	CNSIs	Steroids	+
21	Li et al. (28)	56/M	fever, headache, paralysis of the lower limbs	neck stiffness Confusion	LESCLs	PLE	W:392(95%) P:1773	ME	Steroids	+
22	Mirian et al. (29)	58/M	paraparesis, blurred vision, confusion	tremor, disorientation	cervical cord	PLE	W:217(97%) P:1773	MEM	Steroids	+
23	Qin et al. (5)	51/M	fever, neck stiffness	confusion, seizure, cognitive decline, hiccups	Us	Us	W:29(LRCHL) P:210	TBM	Steroids IVIG	+
24	Qin et al. (5)	66/F	fever, neck stiffness headache, weakness of limbs	dysuria, confusion, hallucinations, balderdash, cognitive decline	DWM/P, BS, BG	Us	W:178 (LRCHL) P:2610	TBM	Steroids	+
25	Qin et al. (5)	65/M	fever, neck stiffness fatigue	dysuria, confusion, hallucinations, balderdash, cognitive decline	BG	Us	W:90(LRCHL) P:1780	TBM	Steroids	+
26	Qin et al. (5)	38/M	fever, neck stiffness headache	weakness of limbs dysuria, confusion, seizure, cognitive decline, fatigue	DWM/P, cervical cord	Us	W:238 (LRCHL) P:1540	TBM	Steroids IVIG	+
27	Qin et al. (5)	51/M	Fever, neck stiffness Headache	dysuria, confusion, hallucinations, balderdash, cognitive decline	subcortex	LME	W:122 (LRCHL) P:2160	TBM	Steroids IVIG	+
28	Wang et al. (30)	28/F	fever, headache, vomiting	seizure, confusion, blurred vision, tremor, leg weakness, memory loss, irritability	cervical cord	PLE	W:300(80%) P:1760	TBM	Steroids	+
29	Wen et al. (31)	68/M	fever, headache	neck stiffness, dysuria, confusion, limb weakness	BS, thoracic cord	BS, T1-T6	W:149(96.6%) P:23563	CNSIs	Steroids IVIG	+
30	Cheng et al. (32)	43/M	fever, headache, vomit	urinary retention, apathy, hiccups, fatigue	the cerebral cortex and hippocampus	PLE	W:310(98%) P:1220	VME	IVIG	+
31	Yaguchi et al. (33)	76/F	fever, dysuria	neck stiffness, confusion	DWM/P, right frontal, parietal lobes	LME	W:130(100%) P:1130	VME	Steroids	+
32	Li et al. (34)	35/F	fever, headache, vomit	Seizure, psychiatric/behavioral abnormalities, cognitive decline	bilateral temporal lobe	PLE	W:34(88%) P:1000	HSVE	Steroids	+
33	Goyne et al. (35)	50/M	fever, vomit, generalised weakness	dysuria, confusion	leptomeningitis, LESCLs	PLE	W:↑(87%) P:3060	ME	Steroids LPEX	+
34	Someko et al. (36)	70/M	fever, headache	confusion	DWM/P	OT	W:23(99%) P:1120	TBM	Steroids	+
35	Wang et al. (37)	16/M	fever, headache	frequent urination, fatigue	SCC	Us	W:20(Us) P:1270	CNSIs	Steroids	+
36	Izumi et al. (38)	59/F	fever, headache	tremor, ataxia, hallucinations, dysuria, confusion	BS, cerebellum	Us	W:126(97%) P:1260	VME	Steroids	+
37	Carneiro et al. (39)	20/F	fever, headache	dysuria, cervicalgia	DWM/P, BS, BG, cerebellum, LESCLs	Us	W:18(LRCHL) P:2535	MEM	Steroids IVIG	+
38	Current case	41/F	fever, headache	confusion, frequent urination, vomit, fatigue, cognitive decline	bilateral thalamus BG, DWM/P, thoracic cord, cervical cord	PLE	W:76(87.3%) P:513	TBM	Steroids	+
39	Current case	74/M	fatigue	tremor, fever, confusion hyperhidrosis	BS, BG, right temporal lobe	LME OT	W:70(95%) P:1256	TBM	Steroids	+
40	Current case	54/M	fever	tremor, confusion, cognitive decline	DWM/P	PLE	W:33(100%) P:807	ME	Steroids	+

Tre, Treatment; Pro, Prognosis; Pri, Primary diagnosis; M, male; F, female; GFAP, glial fibrillary acidic protein; CSF, cerebrospinal fluid; FLAIR, fluid-attenuated inversion recovery; CE-T1WI, contrast enhancement; Tre, Treatment; Pro, Prognosi; Pri, Primary diagnosis; PWM, periventricular white matter; DWM/P, Deep white matter/periventricular; SGM, Subcortical grey matter; BS, Brainstem; SCC, the splenium of the corpus callosum; BG, basal ganglia; LETM, Longitudinal extensive transverse myelitis; LESCLs, longitudinally extensive spinal cord lesions; LRCHL, Lymphocyte predominance type; N,no lesion; IM, improve; Us, Unspecified; CCE, central canal enhancement; LE, linear enhancement; PLE, Perivascular linear enhancement; LME, leptomeningeal enhancement; DSE:diffuse and segmental enhancement; OT, Other; punctate/patchy; TBM, Tuberculous meningitis; MEM, meningoencephalomyelitis; ME, Meningoencephalitis; HSVE, Herpes Simplex Virus Encephalitis; CNSIs, central nervous system infection; ADEM, acute disseminated encephalomyelitis; VME, viral meningoencephalitis; PLEX, plasma exchange; IVIG, IV immunoglobulin; MTX, methotrexate; CTX, Cyclophosphamide; MMF, mycophenolate mofetil; P, protein; WBC, white blood cell, +,improvement of symptoms.

Patients with A-GFAP-A mostly occurred in adulthood, with an average age of onset at 44.75 ± 16.647 years. The male-to-female ratio was 1.08:1 (26 males and 24 females). Among the 40 initially misdiagnosed cases, 14/40 (35%) were misdiagnosed as tuberculous meningitis (TBM) and received anti-tuberculosis treatment; 7/40 (17.5%) were misdiagnosed as central nervous system infections; 1/40 (2.5%) was misdiagnosed as acute disseminated encephalomyelitis (ADEM); 2/40 (5%) were misdiagnosed as herpes simplex virus encephalitis(HSE); 1/40 (2.5%) was misdiagnosed as prion disease; and the remaining 12/40 (30%) were initially diagnosed with clinical manifestations or symptoms and received anti-infective or antiviral treatment. The most common clinical phenotype among the 40 patients was meningitis (50%), followed by meningoencephalitis (22.5%), meningoencephalomyelitis (15%), encephalitis (7.5%), and encephalomyelitis (5%). The main reported clinical symptoms were fever (92.5%), headache (72.5%), altered level of consciousness (65%), neck stiffness (40%), and movement disorders (50%), including gait disturbance, ataxia, tremors, and limb weakness. Autonomic dysfunction (52.5%), including urinary or intestinal dysfunction, excessive sweating, and changes in blood pressure. 32.5% of patients experienced cognitive impairment, including memory loss, disorientation, and other symptoms. In 30% of patients, obvious mental and behavioral symptoms such as hallucinations, gibberish, irritability, panic attack, were observed; 17.5% of patients experienced visual impairment. 39/40 cases (97.5%) showed partial or complete remission after corticosteroid treatment, while 1/40 cases (2.5%) showed significant improvement after intravenous immunoglobulin treatment.

All cases tested positive for GFAP IgG in CSF, and 15/40 cases (37.5%) were tested positive for GFAP IgG in serum, and the most common method was CBA. All cases showed an elevated cell count in CSF. Based on available data, 26 out of 27 cases (96.3%) had lymphocyte count accounted for more than 80%. The protein level in CSF increased, with 31/36 cases (86.1%) showing protein levels greater than or equal to 1.0 g/L, 3/36 cases (8.3%) with protein levels between 0.5 g/L and 1 g/L, and 2/36 cases (5.6%) with protein levels less than 0.5 g/L. In this study, 35/40 cases (87.5%) showed high signal intensity on T_2_/FLAIRimaging; 30/40 cases (75%) had abnormal high T_2_ signals in the brain, including 10/40 cases (25%) in the brainstem, 3/40 cases (7.5%) in the cerebellum, 3/40 cases (7.5%) in the thalamus, 7/40 cases (17.5%) in the basal ganglia, 13/40 cases (32.5%) in the periventricular white matter, 7/40 cases (17.5%) in cerebral lobes, and 4/40 cases (10%) in the corpus callosum; 13/40 cases (32.5%) of patients exhibited abnormal T_2_ signals in the spinal cord, of these, 6/40 cases (15%) had longitudinally extensive abnormal T2 signals, 6/40 cases (15%) showed abnormal T2 signals in the cervical spinal cord, and 2/40 cases (5%) had abnormal T_2_ signals in the thoracic spinal cord. 29/40 cases (72.5%) exhibited abnormal enhancement on T1 signals, of which 15 cases showed perivascular enhancement (37.5%) and 10 cases showed leptomeningeal enhancement (25%) on T1 signals. 5/40 cases (12.5%) exhibited abnormal enhancement in other brain regions, consistent with findings in previous studies (40).

## 4 Discussion

In this study, we report three patients with A-GFAP-A who were initially misdiagnosed with intracranial infections, including two males and one female. All three patients had subacute onset, mainly presenting with symptoms of meningitis such as headache, fever, and changes in consciousness level. The CSF showed an increase in white blood cell count, mainly lymphocytes, elevated protein levels, and decreased glucose levels. These patients were initially misdiagnosed as having viral or tuberculous meningitis. Although the results of PPD, T-SPOT, and CSF pathogen testing in the three patients did not support the diagnosis of tuberculous meningitis, their symptoms continued to deteriorate after anti-infective or antiviral treatment. Unable to rule out the tuberculous meningitis, empirical anti tuberculous treatment was given. However, due to the ineffective anti tuberculous treatment, subsequent positive GFAP IgG testing indicated the diagnosis of A-GFAP-A. After corticosteroid treatment, symptoms improved significantly, making the A-GFAP-A as the more likely diagnosis. Given the lack of a standard treatment protocol for A-GFAP-A, we administered steroids at varying doses based on the severity of the disease. Fortunately, three patients showed rapid improvement after steroid therapy, and two of them received maintenance treatment with mycophenolate mofetil in the later stage. During the follow-up period, two patients had stable disease without recurrence, and one patient had recurrent fluctuations in symptoms. We summarized 40 patients with A-GFAP-A, who presented with acute or subacute meningitis mimicking tuberculous meningitis, and analyzed their clinical, immunological, and imaging characteristics. According to our research and analysis, A-GFAP-A, which is easily misdiagnosed as intracranial infection, can occur at any age and is most likely to start in middle age, with no significant gender difference. The clinical phenotype of patients is mostly manifested as meningitis (50%), followed by meningoencephalitis (22.5%) and meningoencephalomyelitis (15%).

Patients with A-GFAP-A most commonly present with non-specific subacute meningitis symptoms (fever, headache, neck stiffness, vomiting), encephalitis (altered consciousness, tremors, mental symptoms, seizures), myelitis (sensory and motor disorders), cerebellar ataxia, and blurred vision (optic disc edema), with meningeal encephalomyelitis and meningoencephalitis being their main clinical phenotypes (1, 9, 40). The clinical characteristics of the three patients with A-GFAP-A reported in this study were similar to those of infectious meningitis. The three cases we reported, as well as all patients initially misdiagnosed as intracranial infection with A-GFAP-A reported from literature search, the main reason for misdiagnosis were related to the presence of meningitis in patients with A-GFAP-A. Among the 40 misdiagnosed cases summarized in this study, 35% (14/40) of patients were initially diagnosed with TBM and treated with anti-tuberculous therapy. The most common symptoms were meningitis manifestations such as fever, headache, and neck stiffness, which had similar clinical manifestations to TBM. TBM often begins with non-specific symptoms such as headache and fever, and may also present focal neurological symptoms such as tremors, muscle spasms, seizures, etc. Therefore, A-GFAP-A is frequently misdiagnosed as TBM in the early stages of the disease. Additionally, patients with A-GFAP-A may respond to a combination of anti-tuberculosis therapy and steroid treatment, leading to misdiagnosis as TBM. In addition to the three patients initially diagnosed with tuberculous meningitis or viral meningitis and treated with anti-tuberculous or antiviral therapy in our report, other researchers have also reported A-GFAP-A initially misdiagnosed as tuberculous meningitis or meningoencephalitis (5, 24). In this study, the incidence of headache and fever in patients with A-GFAP-A initially misdiagnosed as intracranial infection was 92.5% and 72.5%, respectively, which was significantly higher than the incidence of headache (63.2%) and fever (52%) reported in previous studies (3). This further supports the idea that meningitis is a common clinical manifestation that leads to the misdiagnosis of A-GFAP-A as a central nervous system infection in early stages of the disease.

Notably, two of the three patients in this study exhibited involuntary tremors, and the incidence of tremors in misdiagnosed cases was 15%. Tremor is the second most common movement disorder symptom in A-GFAP-A, typically presenting as postural and/or action tremors (41). The pathogenesis of tremor in A-GFAP-A remains unclear, but increasing evidence suggests that disruption of astrocyte function is linked to dopaminergic neuronal degeneration in Parkinson’s disease. Degeneration of dopaminergic neurons in the substantia nigra is thought to be a neuroinflammatory process in which reactive astrocytes play a key role, with several Parkinson’s disease-associated genes influencing astrocyte activation (42). Disruption of normal inflammatory signaling pathways, potentially triggered by anti-GFAP antibodies, could alter astrocyte function in maintaining neuronal health, leading to dopaminergic neuronal degeneration. Additionally, metabolic dysregulation of neurotransmitters, increased neuronal excitability, and abnormalities in the neural circuitry of the basal ganglia may be involved in the development of the disease. Therefore, GFAP astrocytosis may also contribute to Parkinson’s disease in patients.

In this study, abnormal signals on T2/FLAIR were observed in 35 patients, with lesions in the meninges, brain, spinal cord, optic nerve, or multiple locations. 75% (30/40) of patients presented with brain lesions, which is consistent with previous reports (89.5% and 74%) (1, 40). Our data indicate that brain damage is common in A-GFAP-A patients who are prone to misdiagnosis. The most common neuroimaging manifestations in patients with A-GFAP-A include T2 abnormal signals in the periventricular white matter, with characteristic features of linear enhancement around blood vessels. The former appears in 36% -75% of cases (1, 8), while the latter appears in 42% -53% of cases (8, 9, 40). In our study, 13/40 patients (32.5%) showed abnormal T2 signals in the white matter around the ventricles, 15/40 patients (35%) showed linear enhancement around blood vessels, 10/40 patients (25%) showed leptomeningeal enhancement, and 5 patients (12.5%) showed enhancement in other brain regions, mainly in lobes and sulci, exhibiting linear, arc-shaped segmental, and spotted enhancement. In the cases we summarized, 13 out of 40 patients (32.5%) had lesions involving the spinal cord, with 6 patients presenting with longitudinal extensive spinal cord abnormalities, 6 patients involving the cervical spinal cord, and 2 patients involving the thoracic spinal cord. These results were significantly lower than previous research findings (49% and 53.3%) (40, 43), suggesting that patients with A-GFAP-A are prone to misdiagnosis as intracranial infections in the early stages of the disease. Therefore, for patients with A-GFAP-A without typical imaging manifestations, who were often easy to be misdiagnosed as other diseases in the early stages of the disease, leading to incorrect treatment. For example, in terms of imaging manifestations, diffuse leptomeningeal enhancement can be observed in both patients with A-GFAP-A and TBM. One of the main imaging features of TBM is enhancement of the skull base meninges, especially in the basal ganglia region, which is more pronounced on the contrast-enhanced imaging (44). In patients with A-GFAP-A, there is also a phenomenon of meningeal enhancement in the basal ganglia area (13). Due to the similar clinical manifestations in patients with TBM and A-GFAP-A, contrast-enhanced brain MRI showing meningeal enhancement can easily be misinterpreted as TBM. However, imaging examinations can still help us to some extent for early differentiation and accurate diagnosis. The lesions and manifestations in patients with TBM and A-GFAP-A have their unique features. For patients with A-GFAP-A, multiple regions can be involved, including the cerebellum, basal ganglia, hypothalamus, periventricular white matter, and meninges; On brain MRI, two-thirds of patients show abnormalities in T2/FLAIR weighted imaging, with the most common high signal lesions located in the basal ganglia region (8); Among these, the most specific imaging manifestation is the linear radial enhancement of periventricular blood vessels on contrast-enhanced brain MRI (1, 9). In contrast, the imaging manifestations of TBM include hydrocephalus, meningeal enhancement, tuberculoma, infarction, and basal ganglia calcification. These distinct imaging features can help clinical staff distinguish between these two different diseases (5).

For the enhancement on brain MRI, A-GFAP-A is often misdiagnosed as steroid responsive chronic lymphocytic inflammatory response with abnormal enhancement of pontine vessels (CLIPPERS). The hallmark neuroimaging feature of A-GFAP-A is typical supratentorial linear radial enhancement, which occasionally also can be seen in the cerebellum; However, research has found that perivascular enhancement in patients with A-GFAP-A is not limited to the periventricular area, and linear radial enhancement of the brainstem can also occur (29). Patients with CLIPPERS are characterized by perivascular punctate and linear enhancement lesions mainly affecting the posterior cranial fossa, especially the brainstem. However, lesions involving the supratentorial brain have also been reported (45); At the same time, the disease responds well to corticosteroid treatment (46). In a case series with A-GFAP-A reported, approximately 20% of patients experience recurrence during steroids tapering (47), and 25% of patients exhibit typical perivascular radial enhancement (9), This makes it difficult to distinguish A-GFAP-A from CLIPPERS based on imaging findings or treatment responses, leading to misdiagnosis. However, there are still differences in the MRI manifestations between the two diseases. For example, lesions in CLIPPERS are mostly nodular or “pepper like” high signal lesions limited to the brainstem and cerebellum, occasionally multiple, symmetrical lesions in the cerebral hemisphere being observed (46). A-GFAP-A manifests as more widespread lesions in the brain and spinal cord. Therefore, for relapses with atypical symptoms or radiological features that are effectively treated with steroids therapy, GFAP antibodies should be completed for the differential diagnosis (7).

In this study, 37 patients underwent CSF examination, and all the patients had elevated cerebrospinal fluid cell counts; in 26/27 cases (96.3%), the ratio of lymphocyte was greater than 80%, and 31/36 cases (86.1%) had CSF protein levels≥1.0 g/L. Previous studies have also shown that the CSF of patients with A-GFAP-A disease typically exhibited an inflammatory pattern, with an increase in white blood cells primarily composed of lymphocytes, elevated protein levels, and in some cases, elevated CSF specific oligoclonal bands (8). However, these findings are non-specific as they also occur in patients with CNS infections and tumors. For example, the cerebrospinal fluid of patients with TBM shows an increase in lymphocytes and protein levels (48), while CLIPPERS can also show an increase in lymphocytes and protein levels. It is difficult to distinguish this disease clinically based on the lymphocytes and protein levels in the CSF. In addition, low CSF glucose is common in TBM, which may be caused by abnormal CSF glucose caused by tuberculosis bacteria in the brain or the consumption of glucose by tuberculosis bacteria themselves. However, as an autoimmune disease, there have also been cases with low CSF glucose in patients with A-GFAP-A (13). Therefore, for patients with A-GFAP-A with inflammatory patterns in CSF, clinical manifestations such as meningitis or meningoencephalitis can easily be misdiagnosed as TBM. Recent reports have documented cases of A-GFAP-A complicated by tuberculous meningitis (TBM) (49). A-GFAP-A is occasionally misdiagnosed as TBM due to overlapping clinical features of the two conditions. However, the relationship between the occurrence and sequence of onset of these two diseases remains unclear. Further research is needed to determine whether immune dysfunction predisposes to infection or if infection leads to immune dysfunction. Therefore, in addition to carefully distinguishing between these two diseases, we must consider the possibility of their coexistence. However, there are some key characteristics of CSF that may be helpful for the diagnosis of A-GFAP-A and avoiding misdiagnosis. According to relevant research reports, the elevated levels of lymphocytes and protein levels in the CSF of patients with TBM and A-GFAP-A are different; In TBM patients, lymphocyte and protein levels in CSF are significantly elevated; However, in A-GFAP-A patients, the levels of protein and lymphocytes in CSF are elevated, showing another phenomenon, that is, lymphocytes are mildly elevated while protein levels is significantly elevated (50). This phenomenon can be compared with TBM, providing a direction for distinguishing between the two diseases. The blood characteristics and inflammatory markers of patients with A-GFAP-A are different from those of TBM patients. Although both A-GFAP-A and TBM have severe fever and headache, patients with A-GFAP-A show negative results in PCT, ESR, and CRP tests (24).

Of course, the diagnosis of A-GFAP-A cannot be made relying on solely the positive GFAP IgG. It is necessary to incorporate clinical manifestations, CSF characteristics of test results, and imaging findings for the differential diagnosis with other related diseases or tumors. Research has shown that GFAP is a potential biomarker for brain injury in many central nervous system diseases. When astrocytes are damaged, GFAP is released into CSF and appears in peripheral blood releasing through the blood-brain barrier. Positive GFAP IgG is not only present in the serum or CSF in patients with A-GFAP-A. It can also present in various diseases such as traumatic brain injury (51), spinal cord injury (52),hypoxic-ischemic encephalopathy (53),acute ischemic stroke (54),multiple sclerosis (55), etc. Positive GFAP IgG also has been reported in central nervous system vasculitis (56),NMDAR encephalitis (57),and primary central nervous system lymphoma (58).Therefore, in clinical practice, for atypical manifestations or even clinical features that need to be interpreted with caution, it is necessary to actively work up for other potential causes. It should be kept in mind that GFAP IgG positivity may also indicate a comorbidity in some cases, and antibody positivity cannot be used as a maker for a definitive diagnosis of A-GFAP-A.

A-GFAP-A can exhibit clinical features similar to MOG antibody associated disease (MOGAD) and AQP4 positive neuromyelitis spectrum disease (NMOSD); It has been reported that MOGAD (59) and NMOSD (60) may initially present with headache, fever, meningeal irritation sign, and significant white blood cell count in CSF before the characteristic symptoms of myelitis or optic neuritis. Therefore, differentiation is necessary in clinical practice. Although A-GFAP-A can lead to longitudinal extensive myelitis, there were few reports of permanent paraplegia or quadriplegia in A-GFAP-A, while AQP4 positive neuromyelitis spectrum disease (NMOSD) can result in permanent paralysis (40). Similarly, compared to AQP4 positive NMOSD and MOGAD, the visual impairment of A-GFAP-A is usually painless and mild (40). In A-GFAP-A, spinal cord lesions are usually longitudinally extensive, but compared to AQP4 positive NMOSD, spinal cord edema and swelling are less common. MOGAD is often characterized by the simultaneous occurrence of bilateral optic neuritis, optic disc edema, transverse myelitis involving the conus, and a milder course of the disease. Although multiple regions can also be involved in A-GFAP-A, simultaneous involvement of the optic nerve, optic disc, and spinal cord are rare in (61). Studies showed other autoimmune antibodies, including AQP4 and MOG antibodies, had been found in A-GFAP-A, indicating antibody overlapping of other demyelinating diseases with A-GFAP-A (9); One patient developing A-GFAP-A one year after being diagnosed with AQP4 antibody positive NMOSD was reported; Another patient was found to be positive for GFAP antibodies several months after being diagnosed with MOGAD (62). Therefore, in clinical practice, we should pay attention to the situation of co-antibodies or comorbidities in A-GFAP-A. Before diagnosing A-GFAP-A and during follow-up, we should carefully work out for other autoimmune diseases to avoid misdiagnosis and miss the best treatment opportunity.

There is currently a lack of a standard medical treatment for A-GFAP-A, and empirical immunotherapy is commonly used in clinical practice. The acute phase of A-GFAP-A is often treated with high-dose corticosteroids or immunoglobulin pulse therapy, while for the maintenance therapy, immunosuppressants is often used. 70% of patients show rapid clinical and imaging improvement after the above treatment, and approximately 12.1% -18% of patients have a relapse that requires prolonged treatment (1, 10, 63). For patients with poor efficacy of single steroids therapy, immunosuppressant such as mycophenolate mofetil or azathioprine should be added as an adjunct therapy to the treatment, and rituximab or cyclophosphamide combined with plasma exchange therapy can also be tried. For patients with high titers of GFAP IgG in CSF or extensive brain parenchymal or spinal cord lesions, long-term glucocorticoid therapy, long-term immunosuppressant maintenance, and repeated plasma exchange therapy can be administered to improve prognosis (64). A small number of patients have poor response to hormone or immunoglobulin therapy, and may experience progressive deterioration or even death. For the critically ill patients, repeated plasma exchange or immunosuppressants may be more effective in the early stage of the disease (16). It has been reported that the use of ofatumumab may be well tolerated and an effective alternative in patients with refractory A-GFAP-A or intolerance to rituximab, but its effectiveness and safety still need further research (65). Most patients have a good prognosis, with about 80% of patients presenting with a monophase disease course. After treatment with glucocorticoids and immunosuppressants, repeat MRI of the brain and spinal cord within 18 months showed a reduction in the extent of the lesion compared to previous results. For patients with a good prognosis, the lesion can completely disappear (10, 40, 63). However, 30% of patients still experience relapse, and a small number of patients still have functional impairments or even death (10).

In summary, this study further confirms the presence of fever, meningeal irritation symptoms, altered consciousness level, elevated CSF inflammatory markers, and CSF protein level >1.0g/L in patients with A-GFAP-A mimicking intracranial infection. Imaging findings include symmetrical lesions of the lateral ventricles, diffuse meningeal enhancement, or perivascular radial enhancement, which may be characteristic features of this condition. Therefore, when patients have clinical symptoms of meningitis or meningoencephalitis, routine biochemical tests of CSF are similar to TBM, but with negative results in pathogen testing and non-specific changes in imaging examination, possibility of A-GFAP-A should considered and early detection of antibody GFAP IgG. For CSF showing an increase in white blood cells mainly composed of lymphocytes, with cerebrospinal fluid protein levels greater than 1.0g/L and accompanied by leptomeningeal enhancement, it is also recommended to complete GFAP IgG testing as soon as possible to confirm the diagnosis and avoid delaying treatment.

## Data Availability

The original contributions presented in the study are included in the article/supplementary material. Further inquiries can be directed to the corresponding authors.
